# Zebrafish fin immune responses during high mortality infections with viral haemorrhagic septicemia rhabdovirus. A proteomic and transcriptomic approach

**DOI:** 10.1186/1471-2164-11-518

**Published:** 2010-09-27

**Authors:** Paloma Encinas, Miguel A Rodriguez-Milla, Beatriz Novoa, Amparo Estepa, Antonio Figueras, Julio Coll

**Affiliations:** 1 Instituto Nacional Investigaciones Agrarias, Dpto. Biotecnología. INIA. Crt. La Coruña, Km. 7, 28040 - Madrid, Spain; 2 Instituto Investigaciones Marinas, CSIC, Eduardo Cabello 6, 36208 Vigo, Spain; 3 Universidad Miguel Hernández, IBMC, 03202 Elche, Spain

## Abstract

**Background:**

Despite rhabdoviral infections being one of the best known fish diseases, the gene expression changes induced at the surface tissues after the natural route of infection (infection-by-immersion) have not been described yet. This work describes the differential infected versus non-infected expression of proteins and immune-related transcripts in fins and organs of zebrafish *Danio rerio *shortly after infection-by-immersion with viral haemorrhagic septicemia virus (VHSV).

**Results:**

Two-dimensional differential gel electrophoresis detected variations on the protein levels of the enzymes of the glycolytic pathway and cytoskeleton components but it detected very few immune-related proteins. Differential expression of immune-related gene transcripts estimated by quantitative polymerase chain reaction arrays and hybridization to oligo microarrays showed that while more transcripts increased in fins than in organs (spleen, head kidney and liver), more transcripts decreased in organs than in fins. Increased differential transcript levels in fins detected by both arrays corresponded to previously described infection-related genes such as complement components (*c3b, c8 *and *c9*) or class I histocompatibility antigens (*mhc1*) and to newly described genes such as secreted immunoglobulin domain (*sid4*), macrophage stimulating factor (*mst1*) and a cluster differentiation antigen (*cd36*).

**Conclusions:**

The genes described would contribute to the knowledge of the earliest molecular events occurring in the fish surfaces at the beginning of natural rhabdoviral infections and/or might be new candidates to be tested as adjuvants for fish vaccines.

## Background

The gene expression changes occurring in the surface tissues of fish after rhabdoviral natural infections have not been described yet. In the most studied infection-by-injection models, internal organs are affected by the infection before surface tissues, while the contrary is expected to occur in the infection-by-immersion models which mimick the natural route of infection.

Under laboratory controlled conditions, trout rhabdoviral infections produce 100% mortalities after infection-by-injection, while only 60-80% wild type [1,2] or 5% rhabdoviral-resistant [3-5] mortalities could be obtained after infection-by-immersion. These results suggested that the natural route of infection is an important determinant for the final outcome of fish rhabdoviral diseases.

Some infection-by-immersion studies using recombinant viruses showed that the fin bases, skin and other fish epithelial surfaces were the earliest sites of replication for rhabdoviral [6] and other viral [7] infections. Furthermore, after viral haemorrhagic septicemia (VHSV) infection, zebrafish showed early hemorrhages in the skin, fin bases, mouth and gills long before dying [8]. The fin bases therefore, were chosen for this study as a representative fish surface tissue and because they were easy and reproducibly harvested. From the fish surface tissues, rhabdoviruses spread to the fish internal organs [6,9]. Therefore, internal organs (spleen, head kidney and liver) were also chosen as potential tissue responders to delayed infection-by-immersion and to compare their responses with those obtained at the fin bases.

The zebrafish *Danio rerio *was chosen because the sequence of their genome is well advanced and ~40 K annotated quantitative polymerase chain reaction (Q-PCR) arrays or partially annotated oligo microarrays are available. Furthermore, zebrafish are susceptible to several rhabdoviruses of fish farmed species [2,10-12], whose infection severity can be modulated by viral dosage and temperature. VHSV was selected among the other rhabdoviruses because of their importance in fish aquaculture and because protection and adaptative immune responses to VHSV-challenges after vaccination have been demonstrated in cold-acclimatized zebrafish [8]. Lower than physiological optimal temperatures induce a delay on fish adaptative immune responses while some of the innate immune responses (complement, phagocytosis, etc) are up regulated [13]. However, little is known about the detailed gene regulation implicated in innate responses in fish when temperature decreases and viral susceptibility becomes maximal, such as it occurs during most rhabdoviral natural outbreaks [1,14].

Therefore, the differential (infected versus non-infected ) protein and immune-related transcript expression of zebrafish fin tissues after VHSV infection-by-immersion were the focus of this work. The results demonstrated the important variability associated with *in vivo *fish experiments. At the protein level and despite variability, VHSV-infection changed the differential expression levels of most fin enzymes of the glycolytic pathway and their cytoskeleton proteins. However, because most immune-related proteins remained undetectable by the proteomic approach, their RNA expression levels need to be also investigated. At the transcriptional level, VHSV-infection increased the differential expression of several fin immune-related genes while decreased those of the internal organs. While these findings shed some light upon the earliest effects of VHSV infection-by-immersion at the molecular level, some of the newly described genes might even be used for adjuvant testing purposes.

## Results

### Infection-by-immersion of cold-acclimatized zebrafish with VHSV

**Figure 1:**
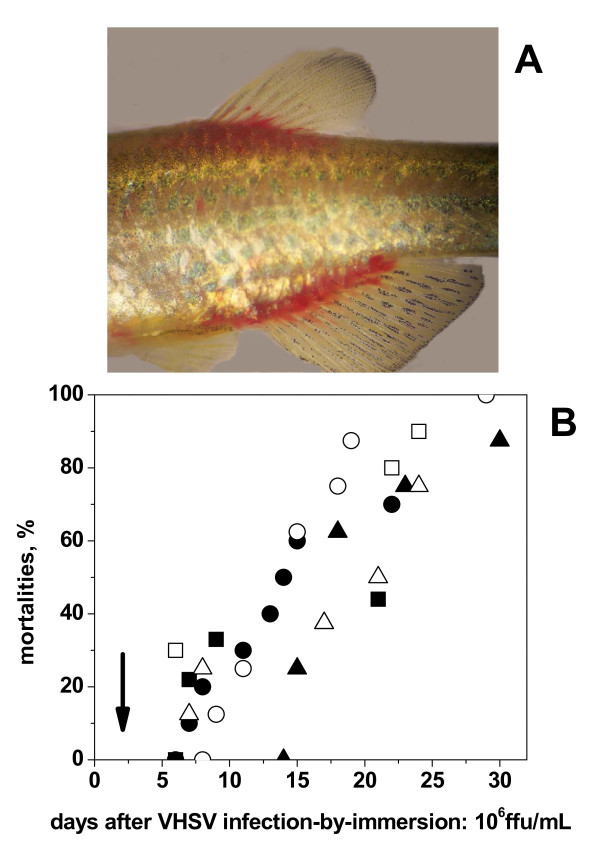
**Photograph of one example of hemorrhages in the fins of VHSV-infected zebrafish (A) and time course of mortalities of VHSV-infected zebrafish (B)**. Adult zebrafish (n = 10) were infected with 2 × 10^6 ^ffu/ml of supernatant from VHSV-07.71 infected EPC cells or virus-free cell cultured medium at 14°C in 50 ml of water. Two hours later they were released into 2 liters aquaria. External hemorrhages were present in all the fish that die. Depending on the fish, hemorrhages first appeared distributed among mouth, lateral or ventral skin and bases of the fins. Figure 1A shows one example which appears in 10-40% of the VHSV-infected zebrafish. Mortalities (Figure 1B) were recorded during the following 30 days in 6 different experiments (different symbols). The vertical arrow indicates the 2-day time at which samples of fins and organs (pooled spleen, head kidney and liver) were harvested for further studies.

Cold acclimatized zebrafish infected-by-immersion in 2 × 10^6 ^focus forming units (ffu) of VHSV per ml, at 14°C induced mortalities of 80-100%. Under those conditions, most zebrafish showed the first external hemorrhages around the mouth and gills, in the lateral skin or in the fin bases, 5-7 days post infection (see one example of external hemorrhages at the fin bases on Figure 1A).

Although every individual zebrafish followed a different time course of VHSV spreading to its body until its death, 2-days after infection-by-immersion there were no mortalities in any of the different experiments performed (n = 6, each experiment consisting of 10 zebrafish). The time courses of subsequent mortalities (Figure 1B) were delayed a few days (50% mortalities occurring 12-20 days after infection) compared to those previously reported in trout (50% mortalities occurring 8-10 days after infection)[15]. Previous zebrafish acclimatation to 14°C during 7 days was required for the VHSV infection to cause high mortalities, confirming previous results [8]. Reduction of the acclimatation time from 7 to 1 days, reduced to 10-30% the mortalities (not shown).

Two days after infection-by-immersion, VHSV N mRNAs levels corresponded to 0.36 × 10^6 ^ffu of VHSV in the fins and 0.007 × 10^6 ^ffu (51.4-fold less) in the organs (pooled spleen, head kidney and liver) per individual zebrafish (averages from 3 experiments, 10 zebrafish per experiment) (data not shown).

Studies on the protein and transcript differential changes occurring on zebrafish fins or organs, 2-days after infection-by-immersion at high mortality conditions were then undertaken.

### Protein changes in the zebrafish fins induced by VHSV infection by 2D-DIGE

**Figure 2:**
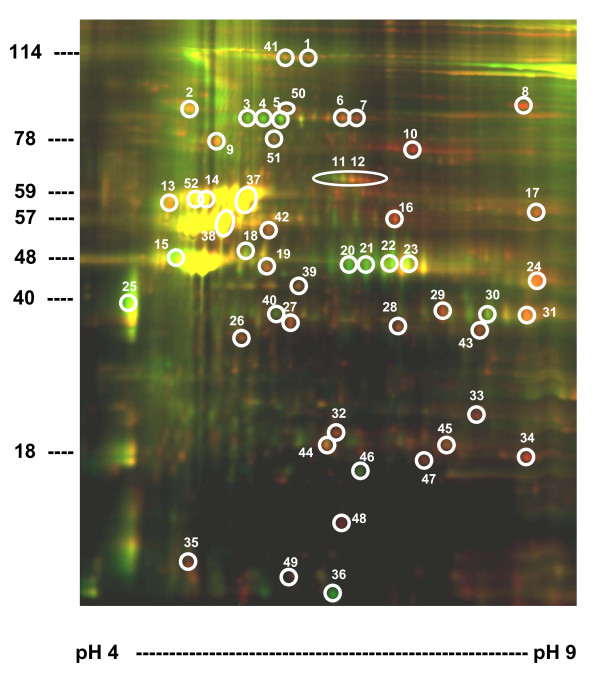
**Representative 2D-DIGE of fin proteins from VHSV- infected (red) and non-infected (green) zebrafish**. Protein extracts from zebrafish fins were analyzed by 2D-DIGE. First dimension was performed on an immobilized pH gradient gel (pH 4 to 9). Second dimension was performed on a 10% SDS-PAGE to separate proteins by their molecular weight (10 to 150 KDa). The circled numbers correspond to the spots which were analyzed by MS. About 70% of the spots analyzed could be identified depending on the experiment (Table 1). Left, molecular weight markers. Bottom, pH gradient from 4 to 9.

Figure 2 illustrates a representative two dimensional differential gel electrophoresis (2D-DIGE) image of comparative protein profiles from VHSV-infected (red) and non-infected (green) fins. About 300 spots in each Sypro Ruby-stained gel were visualized (data not shown). Depending on the experiment, 50-100 spots, that may represent different proteins, degrees of protein modification and/or degradation products were differentially expressed.

The characteristic protein profile of zebrafish fins showed three major multiple spot regions between 48-57 KDa distributed around isoelectric points (pI) of ~5. Their yellow color confirmed that their levels remained unchanged after VHSV infection. Most of the differentially expressed protein spots (decreased in green or increased in red) were between 18 and 78 KDa. The most differentially expressed spots (circled spots on Figure 2) were excised, in-gel digested with trypsin, and analyzed by mass spectrometry (MS). Protein identifications summarized in Table 1 showed that the characteristic major yellow spots corresponded to keratins (~57 KDa), cytokeratins (~52 KDa) and actins (~42 KDa) (Figure 2).

**Table 1:** Differential expression of proteins from fins by 2D-DIGE (Exp1, Exp2 and Exp3) and of their corresponding transcripts by hybridization to oligo microarrays

Spot n°	Protein Name	Accession Number	**~MW ×10**^**-3**^	~pI	fold Exp1	fold Exp2	fold Exp3	microarray
**SIGNALING**								
47	hmgb1	42476233	16	7.0	**2.4**	*3.7	**---**	**1.01**
26	gnb1 (G prot), βpolypep1	47087315	35	5.6	**2.6**	**3.0**	**---**	**1.03**
**APOPTOSIS**								
27	Annexin A1a	31419751	40	6.0	**2.3**	**9.1**	**---**	**1.01**
39	Annexin 1a	27762256	44	6.2	**2.5**	**---**	**---**	**N.F**.
0	Annexin A5b	41107552	36	5.2	**---**	**---**	**-2.0**	**-1.08**
0	Annexin 5b	160773369	36	5.3	**---**	**---**	**-1.9**	**N.F**.
**DETOXIFICATION**								
34	Glutathion S-Transferase	18858197	16	8.2	**11.0**	*2.7	**---**	**1.30**
32	Glutathion S-Transferase	47086689	20	6.4	**4.0**	*2.1	**---**	**1.30**
**HAEMATOPOIESIS**								
10	Transferrin	51859259	70	6.9	**16.1**	*4.6	**---**	**2.40**
11	Hemopexin	33991748	65	6.3	**2.5**	*3.2	**---**	**5.42**
12	Hemopexin	33991748	65	6.5	**3.5**	**---**	**---**	**5.42**
**GLYCOLYSIS & ATP**								
24	Aldolase fructose-bisphosphate	37595414	44	8.3	**5.7**	**8.2**	*1.4	**1.15**
45	Triosephosphate isomerase 1b	47271422	18	7.1	**2.1**	*1.5	**1.5**	**1.00**
29	GAPDH	56718619	42	7.2	**5.3**	**10.2**	*1.8	**1.12**
31	GADPH	53733367	40	8.2	**4.2**	**8.7**	**---**	**1.12**
4	GAPDH	53733367	85	5.6	**-7.4**	**---**	-*1.7	**1.12**
16	Alpha enolase 1	37681795	57	6.7	**6.0**	**17.6**	**1.8**	**1.18**
28	Ldhb Lactate dehydrogenase	28277619	35	6.8	**3.3**	**---**	*2.2	**1.04**
33	Carbonic anhydrase	18858379	22	7.8	**10.4**	**---**	**---**	**-1.04**
43	Malate dehydrogenase	47085883	40	7.8	**2.1**	*1.7	**---**	**1.05**
3	ATP binding	51571925	85	5.5	**-19.1**	**---**	**---**	**-2.22**
0	ATP synthase	41152334	24	7.0	**---**	**---**	**1.5**	**1.05**
46	Creatine kinase	55716037	15	6.5	**-3.4**	**---**	-*1.7	**3.16**
20	Creatine kinase	55716037	48	6.4	**-3.9**	**---**	-*2.0	**3.16**
5	Creatine kinase	55716037	90	5.9	**-4.0**	**---**	-*2.0	**3.16**
**CYTOSKELETON & RELATED PROTEINS**								
42	Keratin 18	41351240	57	5.3	**3.2**	**11.2**	**---**	**1.08**
38	Keratin type I	50370316	57	5.6	**2.4**	**---**	**---**	**1.09**
13	Type II cytokeratin	18858425	59	5.1	**3.2**	**1.0**	**---**	**1.23**
50	Tubulin, gamma assoc protein 2	41056243	85	6.0	**2.4**	**---**	**---**	**-1.01**
30	Skeletal alpha-actin (S. aurata)	6653228	38	7.9	**-2.7**	**-2.8**	**---**	**-1.53**
21	Alpha-tropomyosin	18859505	44	6.6	**---**	**3.4**	**7.2**	**1.56**
0	Alpha-tropomyosin	55962544	36	5.0	**---**	**---**	**11.7**	**1.56**
15	Transgelin	37681953	49	5.2	**-7.4**	**---**	**---**	**1.49**
22	Kinesin-like protein	50055013	50	6.7	**-2.7**	**---**	**---**	**-2.5**
41	Myosin VIa	10116291	114	5.9	**2.0**	*5.1	**7.7**	**1.05**
0	Myosin, light chain 2	18859049	22	5.0	**---**	**---**	**7.3**	**1.30**
19	CapG	29612467	48	5.3	**2.8**	*7.6	**---**	**-1.08**
0	Actin capping protein	41053959	34	5.7	**---**	**---**	**-1.6**	**1.09**
6	Scinderin	42542770	80	6.4	**4.1**	**---**	**---**	**-1.14**
7	Scinderin	42542770	80	6.5	**3.8**	**---**	**---**	**-1.14**
17	Cyclase-associated protein-1	37725381	58	8.3	**5.0**	**---**	**---**	**1.66**
35	Dynamin 1-like	41055508	5	5.3	**3.0**	**---**	**---**	**1.01**
48	Semaphorin 3Gb	57790316	7	6.4	**3.0**	**---**	**---**	**1.09**
14	CkII protein	39645432	65	5.3	**2.9**	**---**	**---**	**1.13**
0	fgf20 fin regeneration	51571925	26	6.2	**---**	**---**	**1.8**	**1.26**
0	fgf20 fin regeneration	51571925	26	6.9	**---**	**---**	**2.2**	**1.26**
0	fgf20 fin regeneration	51571925	26	7.0	**---**	**---**	**2.3**	**1.26**
0	fgf20 fin regeneration	51571925	26	7.2	**---**	**---**	**1.8**	**1.26**

**Exp1**, experiment 1. **Exp2**, experiment 2. **Exp3**, experiment 3. **Spot n°**, number of spot selected in the 2 D gel of Exp1 (Figure 2) for mass spectrophotometry (MS) identification. 0, numbers of spots selected in 2 D gels of exp 2 or 3 (not shown). Gel spots were digested with trypsin and their peptides identified by MS. The corresponding detected proteins with a 100% of probability score of identification are shown grouped by their function. *, spots hypothetically identified by their apparent molecular weight (MW) and isoelectric point (IP) in their respective 2 D gels. **---**, spots too faint to be quantified. **~MW **and **~PI**, apparent molecular weight (MW) and isoelectric points (IP) in the 2 D gel. **Accession number **of the identified protein sequences in the protein bank. **Fold**, calculated by the DeCyder-differential in-gel analysis software by the formula, volume of the spot in the VHSV-infected fin/volume of the spot in the non-infected fin. The spots on Figure 2 which have no corresponding description on Table 2 could not be identified by MS. Microarray differential expression data was obtained as explained in the legend of Table 3. Because most of the protein genes appeared several times in the microarray, their corresponding folds were calculated as the means for each of their data (n = 3-10). **NF**, not found annotated in the microarray.

Most of the enzymes of the glycolytic pathway increased upon VHSV infection, such as fructose-bisphophate aldolase, triose phosphate isomerase, glyceraldehyde 3-phosphate dehydrogenase (*gadph*), phosphopyruvate dehydratase (enolase) and lactate dehydrogenase. Also an ATP binding protein was decreased, while an ATP synthase was increased, most probably contributing with all the above enzymes to ATP accumulation in the VHSV-infected fins (Table 1).

The levels of different types of keratins specific for epithelial cells were maintained after VHSV infection (characteristic yellow spots mentioned above) while others such as keratin 18, type I and type II cytokeratins increased. Skeletal α-actin, α-tropomiosin, kinesin and filament-actin (F-actin) crosslinking transgellin decreased after VHSV infection suggesting the reduction of F-actin networks. Myosins, CapG, scinderin, cyclase-associated protein-1, dynamin and semaphorin also changed after VHSV infection.

Annexins (proteins related to apoptosis) A1a and 1a increased while annexins A5b and 5b decreased with VHSV infection. Proteins implicated in haematopoiesis such as transferrins and hemopexins increased, as probably demanded by the hemorrhages caused by VHSV infection. Increase of 4 isoforms of *fgf20*, a protein implicated in fin regeneration were also detected in one of the experiments (Table 1).

VHSV proteins were searched among those spots located in their expected molecular weight/pI regions on the 2 D gels. However, none could be detected, most probably due to their relative low abundance among the fin proteins 2-days after infection.

Among the few immune-related proteins which could be detected by this approach, only the high mobility group binding 1 (*hmgb1*, spot 47) protein and the guanine nucleotide binding protein β polypeptide 1 (*gnb1*, spot 26) could be detected. Both increased after VHSV infection. Because of the scarce number of immune-related proteins detected by the 2D-DIGE experiments, most probably due to their low concentrations in fins, studies on the VHSV-induced transcripts by using immune-related Q-PCR arrays and hybridization to oligo microarrays were then undertaken.

### Immune-related transcripts changing in fins and organs after VHSV infection analyzed by Q-PCR arrays

**Figure 3:**
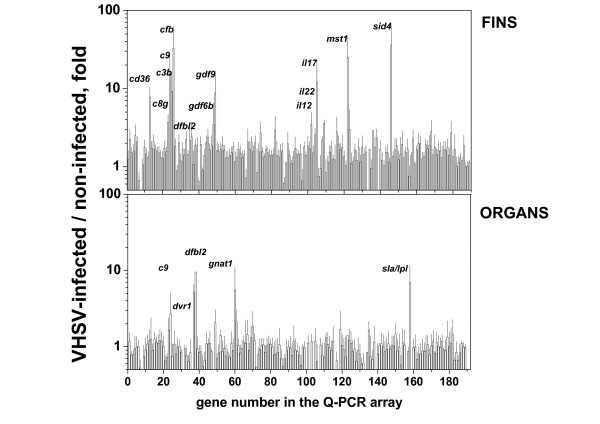
**Differential expression of transcripts from fins and organs detected by the Q-PCR array**. The predesigned Q-PCR TaqMan Assays (Applied Biosystems) targeting 186 immune-related genes from zebrafish were used to estimate their differential transcript levels. Assay conditions and analysis were as described in methods and Table 2. The Figure shows the means and standard errors (SE) from 5 experiments for the 186 immune-related transcripts. Short names in italics of the most important genes are to the left of the bars. The mean values >2 fold and p < 0.05 are in Table 2.

Differential expression with >2-fold and p < 0.05 estimated by Q-PCR arrays showed that while 17 immune-related transcripts increased in fins, only 2 (*c9 *and *sla/lpl*) increased in organs (Table 2). Furthermore, maximal differential transcript levels of any p value were of ~36-fold in fins, while only of ~10 fold in organs (Figure 3).

**Table 2:** Differential expression of transcripts from fins and organs by Q-PCR arrays.

			fold	
			
accession number	**shortname**	long name transcripts	Fins	Organs
**complement components**				
NM_001024435	*c9*	complement component 9	**15.33**	**2.71**
NM_131243	*c3b*	complement component c3b	**9.24**	**-1.30**
NM_200863	*c8g*	complement component 8. gamma polypeptide	**3.69**	**1.63**
**immunoglobulin-related proteins**				
NM_001034182	*sid4*	secreted immunoglobulin domain 4	**35.99**	**1.09**
**cluster differentiation antigens**				
NM_001002363	*cd36*	CD36 antigen	**7.68**	**1.26**
NM_212619	*cd9*	CD9 antigen (p24)	**2.09**	**1.25**
**interleukins**				
NM_001020792	*il22*	interleukin 22	**3.59**	**-1.32**
NM_001020789	*il17d*	interleukin 17d	**2.20**	**1.04**
**major hystocompatibility complex**				
NM_131471	*mhc1uba*	major histocompatibility complex class I UBA gene	**3.01**	**1.41**
**guanine nucleotide binding proteins**				
NM_213224	*gnl2*	guanine nucleotide binding protein-like 2 (nucleolar)	**2.72**	**-1.09**
NM_001002397	*gng7*	guanine nucleotide binding protein gamma 7	**2.02**	**1.21**
**Various**				
NM_152980	*mst1*	macrophage stimulating 1 hepatocyte growth factor	**25.02**	**-1.36**
NM_131607	*tradd*	tnfrsf1a-associated via death domain	**2.83**	**1.07**
NM_001110278	*acvr2a*	activin receptor IIa	**2.49**	**-1.01**
NM_001039637	*foxp1b*	forkhead box P1b	**2.37**	**1.09**
NM_200154	*sla/lpl*	soluble liver antigen/liver pancreas antigen. like	**2.28**	**2.10**
NM_131000	*alcam*	activated leukocyte cell adhesion molecule	**2.25**	**-1.29**
NM_001040353	*crfb12*	cytokine receptor family member B12	**1.68**	**-3.33**

For each experiment, 10 zebrafish were infected with 2 × 10^6 ^ffu of VHSV/ml at 14°C while other 10 zebrafish remained non-infected. RNA was extracted from the fins or organs 2-days after VHSV or mock infection, converted to cDNA and used for the AmpliTaq reaction in 384 well plates containing 2 × 186 selected immune-related zebrafish TaqMan Assays (Applied Biosystems). In each plate, 2 × 3 assays of the *rplp0 *gene were used for normalization. The mean and their corresponding p (t Student one tail) were then calculated from 5 experiments. The transcripts from either fins and/or organs with >2-fold and p < 0.05 changes were first tabulated and then the rest of the table was completed with their corresponding calculated fold values in organs and/or fins, respectively. Fold, expression level in VHSV-infected tissue/expression level in non-infected tissue. +, increased. -, decreased. ~, similar. Figure 3 shows the representation of means and standard errors (SE) of all the genes.

Complement component fin transcripts corresponding to *c3b, c8g *and *c9 *increased after VHSV infection (Table 2), while the same transcripts did not increased in organs, except *c9 *(Table 2).

Major histocompatibility complex class I (*mhc1uba*) transcripts, one of the induced host molecules identified in most infections-by-injection, increased more than 3-fold in the fins, however, it was not increased in organs (Table 2). Other differentially increased transcripts in fins which were not increased in organs were those corresponding to the secreted immunoglobulin domain 4 (*sid4*) of the immunoglobulin superfamily, and macrophage stimulating factor (*mst1*). Among all the cluster differentiation (*cd*) antigen transcripts present in the immune-related probes (*cd2, cd247, cd36, cd63, cd9*), only *cd36 *and *cd 9 *were differentially increased in fins but not in organs (Table 2).

Among the transcripts corresponding to the interleukin (IL) genes studied (*il1b, il10, il12a, il15, il17a/f1. il17a/f2, il17a/f3, il17c, il17 d, il22 *and *il26*), only *il17 d *and its related *il22 *(both produced by T17 helper cells) were differentially increased in fins. Some of the other *il17 *family members such as *il17a/f1 *and *il17 d *were also increased in fins, however, with p > 0.05 (not shown). There were not similar increases in organs for any of the studied IL transcripts (Table 2 and Figure 3).

Interferon (*ifn1*) transcripts, which increased > 2-fold in zebrafish organs after VHSV infection-by-injection in a previous report [8], did not changed at the fins/organs after infection-by-immersion (1.11/-1.44-fold, respectively). However, the primers designed to estimate *ifn1 *by the Q-PCR gene expression assay (Dr03100938_m1), only amplified the virus-independent *ifn1 *isoform which remains unaltered upon viral infection [16].

Defensin β (*defbl2*) increased in organs and fins (Figure 3) but it was eliminated from Table 2, because both of their transcript levels showed p > 0.05.

No immune-related gene transcripts decreased > 2 fold in fins (Table 2), while of the 7 transcripts that decreased > 2-fold in organs, only *crfb12 *have p < 0.05 (Table 2).

### Immune-related transcript changes in fins/organs after VHSV infection by hybridization to oligo microarrays

The signal intensities obtained after hybridization of samples from zebrafish fins and organs to microarrays, ranged from 0 to ~550.000 fluorescence units. Differential expression changes were then calculated by comparing VHSV-infected versus non-infected samples. Of the 43803 oligo sequences present in the zebrafish microarray latest version, 1175 (2.68%) or 730 (1.66%) in fins or organs, respectively, showed > 3-fold increases (original data deposited in GSE19049). On the other hand, 14.78% or 11.70% in fins or organs, respectively showed a significant departure from the null hypothesis when t-tests were performed at the p < 0.05 significance level. Of the 636 immune-related sequences annotated in the zebrafish microarray, 35 (5.5%) in fins and 7 (1.1%) in organs increased > 2-fold with p < 0.05 after VHSV infection. In contrast, 2 (0.3%) sequences in fins and 23 (3.6%) in organs decreased > 2-fold after VHSV infection (Table 3).

**Table 3:** Differential expression of transcripts from fins and organs by hybridization to the oligo microarray

			fold	
			
accession number	*short name*	long name transcripts	Fins	Organs
**complement components**				
NM_131243	*c3b*	complement component c3b	**8.37**	**-1.21**
P01024	*c3*	complement c3 precursor	**6.63**	**1.81**
NM_001024435	*c9*	complement component 9	**5.95**	**1.46**
TC319537	*c9*	complement component 9	**5.85**	**3.33**
TC360850	*c9*	complement component 9	**5.83**	**1.75**
NM_131338	*cfb*	complement factor b	**5.60**	**1.00**
XR_029814	*cfb/c2b*	~to complement factor b/c2b	**5.14**	**-1.27**
NM_001003496	*c8a*	complement component 8, alpha polypeptide	**4.94**	**-1.26**
NM_200863	*c8g*	complement component 8 gamma polypeptide	**1.41**	**-4.34**
TC331566	*crpp*	complement regulatory plasma protein	**4.74**	**-1.11**
XM_0013357	*cfhp*	~to complement factor h precursor	**3.96**	**1.02**
NM_200638	*c6*	complement component 6	**2.22**	**1.11**
XM_692828	*perf*	~to perforin	**-1.78**	**2.93**
**immunoglobulin-related proteins**				
AY494978	*sid4*	secreted immunoglobulin 4 precursor	**5.68**	**1.18**
NM_001034182	*sid4*	secreted immunoglobulin domain 4	**5.30**	**1.26**
AF273876	*ighv4-6*	VH101 immunoglobulin heavy chain variable region	**2.57**	**1.33**
AF273880	*ighv1-2*	VH114 immunoglobulin heavy chain variable region	**2.42**	**1.96**
AF273901	*ighv1-1*	VHcd9 immunoglobulin heavy chain variable region	**2.08**	**3.63**
AY646264	*ighv1-1*	immunoglobulin zeta heavy chain	**2.29**	**1.21**
AY643752	*ighz*	immunoglobulin Z heavy chain constant region	**1.35**	**2.02**
XM_690081	*iefr*	~to immunoglobulin epsilon Fc receptor IgE	**2.64**	**-1.56**
XM_001920109	*pirp*	~to Polymeric immunoglobulin receptor precursor	**2.33**	**1.22**
NM_001145630	*lrit3*	leucine-rich repeat Ig-like and transmembrane domains 3	**-2.85**	**-3.25**
XM_001919777	*npcid*	containing immunoglobulin domains	**5.07**	**-2.07**
**cluster differentiation antigens**				
NM_001002572	*cd36*	CD36 antigen	**6.12**	**-11.11**
Q3U490	*cd11c*	CD11c dendritic cells	**2.53**	**-1.05**
**macrophages**				
NM_152980	*mst1*	macrophage stimulating 1 hepatocyte growth factor	**6.42**	**1.20**
NM_152980	*mst1*	macrophage stimulating 1 hepatocyte growth factor	**5.54**	**1.08**
NM_001113641	*mict*	~to macrophage-inducible C-type lectin	**4.93**	**1.23**
**interleukins**				
Q494Q4	*il11b*	interleukin-11b.	**4.27**	**2.88**
NP_998009	*il1b*	interleukin 1, beta	**2.36**	**1.37**
NM_001020789	*il17d*	interleukin 17d	**-2.27**	**-4.85**
NM_001018118	*il15l*	interleukin 15 like (il15l) transcript variant 2	**-1.07**	**-2.44**
NM_001007108	*il12ba*	interleukin 12Ba natural killer cell stimulatory factor 2	**1.53**	**-25.70**
NM_153660	*il17rd*	interleukin 17 receptor D	**1.04**	**-1.45**
**tolls & receptors**				
Q6TQH6	*tlr7*	toll-like receptor 7	**4.09**	**1.22**
AY389449	*tlr5a*	toll-like receptor 5a	**2.03**	**1.28**
NM_001130594	*tlr9*	toll-like receptor 9	**1.05**	**-2.29**
NM_131010	*tll1*	tolloid-like 1	**-1.72**	**2.82**
NM_001113602	*crlf1b*	cytokine receptor-like factor 1b	**-1.72**	**-2.90**
NM_200600	*traf3ip1*	TNF receptor-associated factor 3 interacting protein 1	**1.19**	**-2.51**
XM_001334668	*traf*	~to TRAF and TNF receptor-associated homolog	**-1.61**	**-2.00**
**major hystocompatibility complex**				
Z46776	*mhc1uaa*	UAA class I MHC	**3.92**	**-23.6**
AJ420954	*mhc1ze*	MHC class I antigen	**2.16**	**1.44**
NM_001045563	*mhc1dz63*	novel MHC class I antigen dZ63M10.3	**-1.14**	**-2.84**
**antimicrobial peptides**				
NM_205583	*hamp1*	hepcidin antimicrobial peptide 1	**10.91**	**-1.04**
NM_205583	*hamp1*	hepcidin antimicrobial peptide 1	**9.63**	**-1.05**
**interferons**				
NM_001111083	*ifn3*	interferon 3	**2.13**	**-1.08**
XM_001344345	*gig2*	~to interferon-inducible protein Gig2	**2.57**	**-2.18**
Q1LVY7	*ifi44*	~to vertebrate interferon-induced protein 44	**1.16**	**-3.30**
**Lymphocytes**				
Q5RI69	*sell*	~to vertebrate selectin L (Lymphocyte adhesion mol 1)	**1.48**	**3.33**
NM_131426	*lef1*	lymphocyte enhancer binding factor 1	**-1.35**	**-5.71**
A1L4S5	*ctlp4*	cytotoxic T-lymphocyte protein 4.	**-1.96**	**-3.26**
XM_001340972	*prf1*	~to Perforin-1 precursor (Lymphocyte pore-forming)	**-1.33**	**-3.07**
NM_001126448	*lect1*	leukocyte cell derived chemotaxin 1	**1.11**	**-2.83**
Q5RJ36	*lnk*	~vertebrate lymphocyte adaptor protein	**-1.08**	**-2.05**
**Various**				
XM_001336492	*sica21*	~to small inducible cytokine A21	**-1.04**	**-3.43**
NM_001030118	*sart3*	squamous cell carcinoma antigen recognised by T cells 3	**0.96**	**-3.39**
NM_001030118	*sart3*	squamous cell carcinoma antigen recognised by T cells 3	**1.13**	**-2.97**

**Figure 4:**
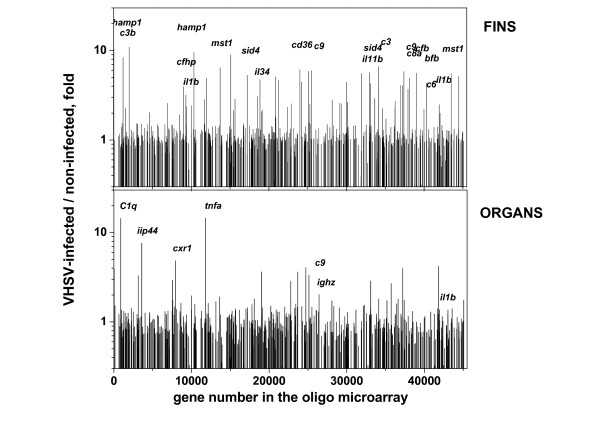
**Differential expression of transcripts from fins and organs detected by hybridization to the oligo microarray**. The selected 636 immune-related annotated genes from the zebrafish oligo microarray (Agilent) were used to estimate their differential transcript levels. Assay conditions and analysis were as described in methods and Table 3. The Figure shows the means from 4 experiments for the 636 immune-related transcripts. Short names in italics of the most important genes are to the left of the bars. The mean values >2 fold and p < 0.05 are in Table 3.

For each experiment, 10 zebrafish were infected with 2 × 10^6 ^ffu of VHSV/ml at 14°C while other 10 zebrafish remained non-infected. RNA was extracted from the fins or organs 2-days after VHSV or mock infection, labeled with Cy3 and hybridized to the microarrays. The 636 immune-related selected sequences from the 4x44 K microarray (Appligene) from zebrafish were selected and their data analyzed. In each microarray, 4 assays of *rplp0 *gene were used for normalization. The mean and p (t Student one tail) were then calculated from 4 experiments. The transcripts from either fins and/or organs with >2-fold and p < 0.05 changes were first tabulated and then the rest of the table was completed with their corresponding calculated fold values in organs and/or fins, respectively. Fold, expression level in VHSV-infected tissue/expression level in non-infected tissue. +, increased. -, decreased. ~, similar. Figure 4 shows the representation of the means of all the immune-related genes.

The genes corresponding to the proteins identified in the proteomic analysis were searched in the microarray data and a column with that data added to Table 1. Because they were present in several copies per microarray, the averages of their differential transcriptional levels were calculated and represented. Although some of the increased (transferrin and hemopexin) and decreased (annexin A5b, ATP binding, alpha actin, and kinesin) proteins showed a similar change in their differential transcript levels, the rest of protein changes did not correlate with those of their corresponding transcripts.

Many complement transcripts (*c3 precursor, c9, cfb, cfb/c2b, c8a, crpp, cfhp*, and *c6*) including those increased in the Q-PCR arrays (*c9, c3b *and *c8g*), increased in fins (Table 3). Only the *c9 *transcripts increased in both fins and organs (Table 3 and Figure 4), as confirmed by 3 different oligo sequences of the same gene present in the microarray. Sequences for the rest of complement related genes (*c2*, *c4*, *c5 *or *c7 *genes) were neither present in the microarray (GSE19049) nor in the Q-PCR array (GSE19503). Increased *c1q *transcripts (antibody-dependent complement pathway) were only found increased in organs but with p > 0.05 (Figure 4).

Of the 29 major histocompatibility complex (*mhc*) sequences present in the annotated immune-related microarray sequences, only those corresponding to the *mhc1uaa *and *mhc1ze *genes in fins (Table 3) were increased. However, most of their lower folds and p > 0.05 values resulted from their higher folds in only one of the experiments (data not shown).

Previously described up-regulated typical gene markers of viral infection-by-injection, *il1b *(interleukin 1 β, >2 fold) or *tnfa *(tumor necrosis factor alpha, >14 fold) were found among the genes increased in fins or organs (Figure 4) but with p > 0.05.

Two different oligo sequences from the hepcidin antimicrobial peptide 1 (*hamp1*) gene detected similarly increased transcripts in the fins but not in organs (Table 3 and Figure 4).

Some of the 64 transcripts corresponding to immunoglobulin-related genes (*ig*), specially those corresponding to the heavy chain gene, increased in the fins (*ighv4-6*, *ighv1-2*, *ighv1-1 *and *ighv1-1zeta*) (Table 3) and in the organs (*ighv1-1 *and *ighz*). Among the transcripts increased, those corresponding to the newly described immunoglobulin Z, both at the fins (*ighv1-1zeta*) and at the organs (*ighz*) might be relevant to mucosal immunity as could be expected from a natural route of infection.

**Figure 5:**
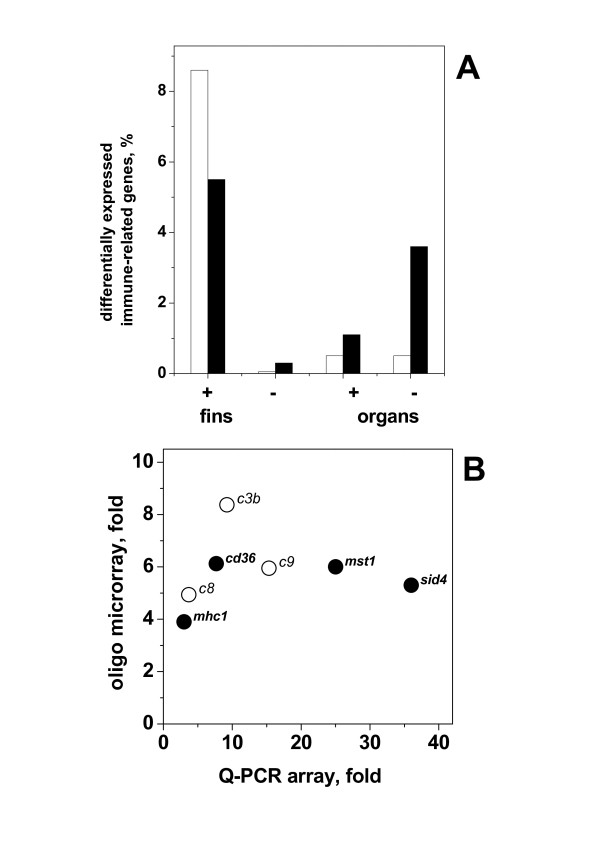
**Total numbers of differentially expressed genes from fins and organs detected by Q-PCR and oligo microarrays (A) and differentially increased fin genes by both Q-PCR and oligo microarrays (B)**. A) Represents the total number of genes increasing > 2-fold with p < 0.05 (Tables 2 and 3) as the percentage of the total number of immune-related genes assayed in Q-PCR (open bars) or oligo microarrays (black bars). Percentages were calculated by the formula, 100 × number of differentially expressed genes/total number of immune-related genes assayed. +, increasing after VHSV infection. -, decreasing after VHSV infection. B) Represents the differential fin transcript levels together with their gene short names that increased >2-fold and p < 0.05 in both Q-PCR and oligo microarrays.

Figure 5B shows the comparison of fold levels between the transcripts that were differentially increased in both Q-PCR array and microarrays. Only *c3b, c8 *(2 different subunits), *c9*, *mhc1 *(2 different genes)*, sid4*, *mst1 *and *cd36 *fin transcripts were increased in both Q-PCR array (Table 2) and microarray (Table 3).

## Discussion

### Major protein changes at the fins after VHSV infection

The differential expression (VHSV-infected versus non-infected) of fin proteins, identified their most abundant proteins with high variations among experiments (Table 1). Individual fish variation in the development of VHSV infection and on the different amounts of muscle sampled at the bases of the fins (10-20%), different yields of the fin protein precipitation techniques and 2D-DIGE technical irreproducibility, all contributed to the wide quantitative differences. Nevertheless, some differential abundances of the major protein components could be detected.

For instance, most of the enzymes of the glycolytic pathway were differentially increased (Table 1). Similar enzymes were also found among the cDNA library genes from HRV-infected leukocytes of japanese flounder [17,18]. In contrast, no enzyme transcripts related to the glycolytic pathway increased in organs after HRV [19] or IHNV [20] infection-by-injection, nor in DNA vaccination-by-injection with the G gene of HRV [21], VHSV [22] or IHNV [23].

Many proteins classified and/or related to the cytoskeleton (actins, dynamin, semaphorin, myosin, etc), vesicle trafficking and/or fin regeneration (*fgf20*) [24] were differentially expressed (Table 1). At least some of those might reflect fin regeneration attempts to compensate for their destruction as VHSV infection progresses. Similar proteins were also found among the cDNA obtained from the HRV-infected leukocytes of the japanese flounder mentioned above [17].

Increases of *hmgb1 *protein levels (2.4-3.7-fold, Table 1) were detected in VHSV-infected fins. However, their transcript levels were unchanged (Table 1). Similarly, *hmgb1 *transcript profiles after intramuscular injection of the IHNV G gene in trout showed that none of their 6 cDNAs microarray probes changed [23]. Nevertheless, transcripts of *hmgb1 *were identified among the HRV-infected leukocytes of japanese flounder [17,18] and a 1.9-fold increase of *hmgb2*-*like 1 *kidney transcripts was found in IHNV-infected trout (Dr. J.C. Balasch, personal communication) (MacKenzie et al., 2008). In mammalians, *hmgb1 *is an abundant nuclear protein being secreted upon bacterial infections to enhance tissue repair, attract inflammatory cells, induce chemokines, and activate dendritic cells. All these properties make *hmgb1 *an important intermediate between innate and adaptative responses [25] and a vaccine adjuvant candidate [26]. Most recently, secreted *hmgb1 *from dendritic cells was demonstrated during Dengue viral replication [27] and it has also been tested as a vaccine adjuvant [28,29]. The potential use of *hmgb1 *as an adjuvant for fish vaccines, therefore, might deserve further attention.

The differential expression of some fin proteins (transferrin, hemopexin, annexin A5b, ATP binding, alpha actin, and kinesin) showed a parallel variation in their transcript levels (Table 1). However, in most of them, the protein differential expression changes did not correlate with their corresponding transcript changes, suggesting that regulation of their expression was not at the transcriptional level, at least in 2-day VHSV-infected fins. Although correlation of gene and protein expression levels have been found in some plants [30,31], most studies found no correlation, including a recent report on individual *E. coli *cells [32]. On the other hand, exclusive post-translational regulation of enzymes of the glycolytic pathway has been reported [33], which could explain some of the data found here. Although, similar studies are still scarce, correlation values comparing gene/protein expression levels in several systems were very low [34], and varied with the protein relative abundance and their half-life time. Most correlation values showed that mRNA might be a poor indicator of protein expression. Study of mRNA levels, is justified when there is no other way to study gene expression levels, as in the present case where the VHSV immune-related proteins cannot be detected by the proteomic approach.

### Transcript level changes of immune-related genes at the fins and organs after VHSV infection

The number of the 186 immune-related transcripts differentially increased were higher in fins than in organs as estimated by Q-PCR. Similar results were obtained with the 636 immune-related transcripts estimated by microarray hybridization. In contrast, the number of differentially decreased transcripts were higher in organs than in fins (Figure 5A). It seems likely that 2-days after infection-by-immersion, VHSV has not yet caused an increased differential response from the internal organs, contrary to what has been described for infection-by-injection in other fish models [17-23,35-40]. The lower numbers of increased transcripts seen in organs compared to fins may be due to a delay in virus tissue infiltration. Thus, the reason for the major differences seen in transcription may be solely because 51.4-fold more VHSV was present in fins than in organs. However, if the decreases in transcription are due to the virus, one would expect to see the most significant decreases in fins where the VHSV is more abundant. As an alternative explanation, transcriptions in organs could be inhibited by some soluble viral protein(s) released from the infected fish surfaces. There are not yet any evidences to decide for an explanation of these data.

The differential increased expression obtained in fins indicate that zebrafish responses are not fully immunocompromised at low temperatures. Low external and therefore low body temperatures in zebrafish can be immunosuppressive, as it has been supported by numerous studies. However, most of those studies demonstrate a delay or inhibition of adaptative immune responses, while at least some innate defenses were increased until the specific immune system adapted [13,41,42]. Thus, cold acclimatized zebrafish fins increased the expression of proteins and transcripts of some immune-related genes after VHSV infection (Tables 1, 2 and 3), confirming some previous results on rhabdoviral vaccinated zebrafish [8]. However, under the conditions used, most of the organ early responses previously described in infection-by-injection models could not be detected in fins (*mx*, *tnfa*, *il8*, *ifn*, etc) [17-23,39,40], although some of them (i.e. *tnfa*) were detected in organs (Table 3 and Figure 4). On the other hand, because VHSV [8] or spring viremia carp virus SVCV [12,14] maximal mortalities only occurred at lower than physiological temperatures, there is no other way to study gene responses to rhabdoviral infections. High mortalities at low temperatures could be explained by a more rapid VHSV replication rate in comparison with slower zebrafish defenses, such as it was suggested in the zebrafish/SVCV model [12]. Comparison of the gene expressions between zebrafish/VHSV and zebrafish/SVCV models might help to identify new genes, since while mortalities are maximal at similar temperatures (12-14°C) [14], optimal SVCV *in vitro *replication occurs at 20-22°C which is closer to the zebrafish optimal temperature. Because zebrafish without cold acclimatation was refractory to infection-by-immersion with IHNV [10], VHSV [8] or even with SVCV [12], there must exist some temperature-dependent host response mechanism(s) that inhibits rhabdoviral infection and/or their spreading. This temperature-dependence might be of interest for further studies.

Figure 5B shows the fin transcripts increased with >2-fold and p < 0.05 in both Q-PCR (Table 2) and oligo microarrays (Table 3). Only complement components (*c3b, c8 *and *c9*), class I histocompatibility antigens (*mhc1*), secreted immunoglobulin domain (*sid4*), macrophage stimulating factor (*mst1*) and a cluster differentiation antigen (*cd36*) were detected in both arrays under those conditions. Transcripts related to the complement pathway (*c3b*) and their corresponding terminal lysis-complex genes (*c8 *and *c9*) [43], were also found in ESTs from HRV-infected leukocytes of japanese flounder [17]. However, many more complement-related genes which were not present in the Q-PCR array, were detected by the oligo microarray data (*c3b*, *c3 precursor, c9, cfb, cfb/c2b, c8a, crpp, cfhp *and *c6*) (Table 3), suggesting that complement components are one of the first lines of defense induced in VHSV-infected fins. In this respect, earlier studies failed to demonstrate a possible relation of trout *c3 *genetic polymorphisms to VHSV resistance [44]. On the other hand, although some reports do exist on exploration of *c3a, c3 d*, *c4a *and *c5a *[45] as vaccine adjuvants in mammals, they remain unexplored in fish. With respect to *mhc *genes, most class I antigens represented in the microarray remained unchanged although high variations among experiments were detected in most genes and a few of them in either fins or organs changed (Tables 2 and 3). At least some *mhc *genes also remained unchanged in trout after IHNV infection-by-injection [46]. The secreted immunoglobulin 4 (*sid4*) of the immunoglobulin superfamily [47] and the macrophage stimulating factor (*mst1*) [48] have been scarcely studied. In mammalians, *cd36 *is restricted to platelets, monocytes, B-cells, macrophages, keratinocytes, epithelial and endothelial cells. On macrophages, *cd36 *is involved in phagocytic clearance of apoptotic cells [49].

Increased *il12 *transcripts with >2-fold but p > 0.05, were found in the fins by both arrays while they were inhibited in organs (not shown). *Il12 *is produced in mammalian macrophages, monocytes, dendritic cells and B lymphocytes in response to intracellular pathogens. *Il12 *stimulates *tnfa*, but no induction of *tnfa *was observed in fins despite of the presence of induced expression levels of *Il12*. Nevertheless, *tnfa *was the most increased transcript in organs according to the oligo microarray data (Figure 3) but with p > 0.05. *Il12 *has been abundantly described as a viral vaccine adjuvant to increase protective mucosal immunity [50]. It has not been tested in fish.

Other transcripts only appeared differentially expressed in one of the arrays, such as *il17 d *and *il22 *(Table 2). Both *il17 *and *il22 *are produced by T helper 17 (Th17) cells [51] to synergistically induce antimicrobial peptides [52] in human keratinocytes [53]. At this respect, 2 antimicrobial peptides increased in VHSV-infected zebrafish fins and organs, including the hepcidin antimicrobial peptide 1 (*hamp1*) and the β defensin (*defbl2*) genes, the former one with p > 0.05. Increased *hamp1 *had also been detected in turbot by microarray analysis of bacterial [54] and nodavirus [55] infections and in stimulated RTS11 trout macrophage cell line [56]. Since, previous findings also implicated *defbl2 *in both rhabdoviral blocking and activation of trout immune defense genes [57], these 2 antimicrobial peptides might deserve further studies.

## Conclusions

VHSV, IHNV and SVCV natural infections with maximal mortalities occur in salmonids and/or carp after low or changing temperatures in the spring and/or autumn. The results described here demonstrate that, at least zebrafish are not fully immunocompromised at low temperatures. Increased fin transcript levels detected by both Q-PCR and hybridization arrays corresponded to previously described infection-related genes such as complement components (*c3b, c8 *and *c9*) or class I histocompatibility antigens (*mhc1*) and to newly described genes such as secreted immunoglobulin domain (*sid4*), macrophage stimulating factor (*mst1*) and a cluster differentiation antigen (*cd36*). The genes described would contribute to the knowledge of the earliest molecular events occurring in the fish surface tissues at the beginning of natural rhabdoviral infections and/or might be new candidates to be tested as molecular adjuvants for fish vaccines.

## Methods

### Viruses and cell culture

The VHSV 07.71 isolated in France from rainbow trout *Onchorynchus mykiss *(Walbaum) was grown in the *Epithelioma papulosum cyprini *(EPC) cells obtained from the ATCC collection (CRL-2872), recently identified as belonging to fathead minnow (*Pimephales promelas*). They were grown in 25 cm^2 ^flasks at 28°C in RPMI Dutch modified cell culture medium buffered with 20 mM HEPES (Flow) and supplemented with 10% fetal calf serum, 1 mM piruvate, 2 mM glutamine, 50 μg/ml of gentamicin and 2.5 μg/ml of fungizone. To prepare VHSV for *in vivo *challenges and to assay for VHSV infectivity, the cell culture media was the same as above except for the inclusion of 2% fetal calf serum and 10 mM Tris pH 8.0. The VHSV was assayed by immunodetection of infected foci in EPC cell monolayers as described before [58].

### Zebrafish infection with VHSV and tissue harvest

Adult zebrafish of 2-3 g (~4 cm in length) were obtained from a local fish pet shop to study a situation that more closely resembles the variability of natural populations. Zebrafish were maintained at 24-26°C in 30 l aquaria with tap-dechlorinated carbon-filtered water with 1 g of CaCl_2, _1 g of NaHCO_3 _and 0.5 g of Instant Ocean sea salts added to water resulting in a conductivity of 200-300 μS pH of 7.8-8.2. The aquaria were provided with biological filters and fish fed with a commercial feed diet. For each experiment, groups of 10 adult zebrafish were moved to 2 l aquaria provided with biological filters at 14°C for cold acclimatation. After 7 days, groups of 10 zebrafish were infected-by-immersion in 2 × 10^6 ^ffu of VHSV per ml at 14°C during 2 h in 50 ml aerated bottles filled with aquarium water. Zebrafish were then released to the 2 l aquaria and maintained during 2-days at 14°C. After decapitation, blood was collected in 100 μl of sterilized anti-coagulation medium (0.64 g of sodium citrate, 0.15 g of EDTA and 0.9 g of sodium chloride in 100 ml of distilled water) per fish, then pooled and centrifuged to obtain 1/10-1/20 diluted plasma (as evaluated by nanodrop absorbance at 280 nm). Fins (dorsal, ventral and caudal) or organs (spleen, head kidney and liver) were harvested and separately pooled from each group of 10 fish to obtain enough protein and RNA to analyze the data. By visual inspection, a 10-20% of muscle tissue was included in the fin samples. The fins or organs were immersed in RNAlater (Ambion, Austin, USA ) at 4°C overnight before being frozen at -70°C until processed. Experimental protocols were performed with the approval of the Departamento Biotecnologia (INIA) and the Instituto de Investigaciones Marinas (CSIC) corresponding ethic committees.

### Sample preparation and labeling with cyanine dyes for two dimensional (2D) differential gel electrophoresis (DIGE)

Fins pooled from 10 zebrafish were sonicated (Braunsonic 300S) in 1 ml of RTL buffer (RNeasy kits from Qiagen, Hilden, Germany) at 10W 1-2 min on ice. The homogenate diluted 3-fold with cold acetone was centrifuged 15 min at 12.000 *g*. Protein pellets were dissolved in water, sonicated and centrifuged again. Water insoluble pellets were discarded and aqueous extract protein concentrations were estimated by the BCA micro protein assay kit (Pierce, Rockford, IL, USA), by nanodrop ND1000 spectrophotometer (Nanodrop Technologies Inc, Wilmington, DE, USA) and confirmed by polyacrylamide gel electrophoresis for accurate normalization.

Thirty micrograms of protein from VHSV-infected and non-infected fins were separately labeled (Appplied Biomics, Hayward, CA, USA) with fluorescent Cy3 and Cy5, respectively (Amersham Biosciences, Inc. Piscataway, NJ, USA) and then pooled. The first dimension of the 2D-DIGE used an immobilized pH gradient gel (Amersham Biosciences, GE Healthcare N.J.). After second dimension SDS-PAGE, the gel was scanned using the Typhoon Trio scanner and images analyzed with ImageQuant software (Amersham Biosciences). Quantification of protein expression was carried out by DeCyder-differential in-gel analysis software (Applied Biomics, Hayward, CA, USA). Protein spots with >2-fold change between VHSV-infected and non-infected fin extracts were excised from preparative gels (~300 μg of protein) by using an Ettan spot picker (Amersham Biosciences) and digested with trypsin.

The trypsin-digested peptides were used for MALDI-TOF protein identification (MALDI-TOF/TOF mass spectrophotometer, ABI-4700 from Applied Biosystems, Inc, Foster City, CA, USA). By using the Mascot search engine (Matrix Science, Boston, MA, USA), the National Center for Biotechnology (NCBI)/SwissProt protein data bases were searched for > 95% matches of high quality mass spectra.

### Quantification of the VHSV N protein mRNA levels by RT-Q-PCR

The primers 5'-TCAAGGTGACACAGGCAGTCA (sense), 5'-CCAGTTCTCTCATGGGCATCAT (antisense) and 5'-CCACGAGCATCGAGGCGGGAAT (labeled with 6-carboxyfluorescein, FAM and 6-carboxy-tetrametil-rodamine, TAMRA) were used to detect VHSV N transcript by quantitative reverse transcriptase polymerase chain reaction (RT-Q-PCR) as described before [59]. Briefly, 50 pg of each primer and the labeled probe were added to cDNA obtained from 25 ng of RNA from VHSV concentrated from VHSV-infected EPC cell cultures (reference curve made from 10^10 ^ffu of VHSV per ml), and zebrafish fins or organ extracts. Then, 4 units of Ampli-Taq polymerase (Perkin-Elmer, Weiterstadt, Germany), buffer and water to 100 μl were added and the mixtures heated to 50°C for 2 min, denatured at 95°C 10 min and then amplified by 45 cycles of 15 seconds at 95°C and 1 min at 60°C. The products were analyzed in a Rotor-gene 2000 machine (RCorbett Research, Sydney, Australia ). The abundance of the corresponding mRNA was calculated from the cycle threshold (Ct) data in 3 different experiments, 10 zebrafish per experiment each by duplicate.

### Selection of zebrafish immune-related genes from Applied Biosystems (Q-PCR) array

The 2008 latest collection of pre-designed, real time PCR assays in the TaqMan^® ^Assays (Applied Biosystems) targeting 49826 sequences of zebrafish https://products.appliedbiosystems.com was searched for immune-related gene keywords: interferon, chemokine, interleukin, cytokine, defensin, macrophage, lymphocyte, antimicrobial, neutrophil, leukocyte, cytotoxic, natural killer, antiviral, antibacterial, LPS, Vig, antigen, cd* antigen, histocompatibility, phagocyte, viral, Mx, complement, immunoglobulin, hepcidin, IgG, IgM, Toll, T cell, B cell, dendritic, presenting, TANK, GNB, HMGB, TNF and MHC. All the entries retrieved were incorporated into an unique archive, duplicates eliminated, primers-probe spanning an exon junction ( _m1) selected and the resulting gene list sent to the TaqMan custom plating service. A total of 186 immune-related genes were included into a 384 well plate (2 × 192 sequences per plate) (GEO platform number GPL9804). Three sets from the ribosomal protein large P0 (*rplp0*) gene (Dr03131549_m1, Dr03131547_g1 and Dr03131546_m1, corresponding to the gene accession number NM_131580.1) were included for normalization purposes as used before [23].

### Quantitative estimation of zebrafish transcripts for selected immune-related 186 gene arrays by Q-PCR

RNA was extracted from sonicated (1 min × 3 times at 40 W in ice) zebrafish fins or organs (pooled spleen, head kidney and liver) in RTL buffer (RNeasy kits, Qiagen, Hilden, Germany). RNA concentrations were estimated by nanodrop and the presence of 18 and 28 S bands confirmed by denaturing RNA agar electrophoresis (Sigma, Che.Co, MS, USA). Three μg of RNA were immediately converted to cDNA by using the High Capacity RNA-to-cDNA Master mix (Applied Biosystems) by 30 min at 42°C. One ng of cDNA was mixed with TaqMan^® ^gene expression assays in a 10 μl final volume and heated to 50°C 2 min (UDG decontaminating step), heat denatured (95°C 10 min) and amplified by 35 cycles of 95°C 15 seconds and 60°C 1 min in a 7900 H Applied Biosystems machine in the FAM channel (470/510 nm). By using 3 TaqMan assays (*hmgb1, gnb1 *and *rplp0*) the amplification conditions were first adjusted to obtain lineal amplifications between 0.01 to 100 ng of RNA (data not shown). Each experiment containing VHSV-infected and non-infected samples was RT-Q-PCR amplified independently during a 5 month period. For each experiment, the relative number of molecules were calculated from the cycle threshold (Ct) data by using the 2^-deltadelta ^relative quantitation method. Raw Ct were normalized for each experiment http://www.ncbi.nlm.nih.gov/geo/query/acc.cgi?acc=GSE19503 by using the *rplp0 *gene [23] according to the formula Ct gene - mean Ct of *rplp0 *(n = 3). Fold for each experiment was then calculated by the 2^-(Ct VHSV-infected-Ct non-infected) ^formula and means and standard errors calculated (n = 5). The p values corresponding to the hypothesis of VHSV-infected > non-infected were calculated by the one tail t-Student test. Outliers (significant results only in one experiment) were identified and eliminated from the calculations manually. Calculations were made by two independent researchers using Microsoft Excel, Origin pro vs 8.0 SR4 (Northampton, USA) and BRB-Array Tools http://linus.nci.nih.gov/BRB-ArrayTools.html. Their results were confronted until all discrepancies were solved.

### Quantitation of zebrafish immune-related transcripts by hybridization to oligo microarray

The 4x44 K format zebrafish 60-mer oligo microarray (zebrafish vs2, ID019161) containing 43803 sequences was obtained from Agilent http://www.chem.agilent.com/en-US/pages/homepage.aspx. Some sequences (40-50%) still contained preliminarily accession numbers while ~10% of their sequences remained unknown at September 2009. Current microarray annotations were searched with the same keywords used for the Q-PCR arrays described above to identify 636 immune-related sequences. Each microarray contained 4 sequences of the *rplp0 *gene that were used for normalization purposes [23]. A total of 16 microarrays grouped in four 4x44 K slides (2 slides for fins and 2 for organs, each containing 2 VHSV-infected and 2 non-infected samples) were used. RNA was kept frozen at -80°C until all the experiments were hybridized and processed simultaneously. Labeling of 2 μg of RNA (~50 μg/ml) and hybridization to the microarrays were performed by the University of Santiago de Compostela at Lugo, Dpt. of Genetics, Spain (Dr.P.Martinez Portela) as described before [54], complying with the Minimum Information About a Microarray Experiment (MIAME) standards. Briefly, high quality RNA were labeled with Cy3 (Amersham Pharmacia) by using SuperScript III reverse transcriptase (InVitroGen) and oligo(dT) primer, and the resulting cDNA was purified with Microcon YM30 (Millipore). The slides were pre-treated with 1% BSA, fraction V, 5 × SSC, 0.1% SDS (30 min at 50°C) and washed with 2 × SSC (3 min) and 0.2 × SSC (3 min) and hybridized overnight in cocktail containing 1.3 × Denhardt's, 3 × SSC 0.3% SDS, 2.1 μg/μl polyadenylate and 1 μg/μl yeast tRNA. Signal was captured, processed and segmented using an Agilent scanner (G2565B, AgilentTechnologies) by the Agilent Feature ExtractionSoftware (v9.5) with the protocol GE1-v5_95, extended dynamic range and preprocessing by the Agilent feature extraction. Normalization within each microarray was carried out by using the mean of tetraplicates of the *rplp0 *gene http://www.ncbi.nlm.nih.gov/geo/query/acc.cgi?acc=GSE19049. The gProccesedSignal was chosen for statistical analysis. Data was first filtered by non-uniform pixel distributed outliers and other replicate outliers (glsFeatNonUnifOL, glsBGNonUnifOL, glsFeatPopnOL and glsBGPopnOL) according to the default Agilent feature extraction criteria; ratio between processed signal and its error <2; differentiation from background signal; linear relationship between concentration and intensity below limits according to Spike-In information and/or, at least 2 quality biological replicates out of 4. The list of sequences was searched for each of the immune-related gene keywords defined above and analysis continued with those selected 636 genes (the rest of the genes will be analyzed and reported elsewhere). For each immune-related gene the Student t one tail statistic associated p was computed and fold calculated by the formula, mean of VHSV-infected values/mean of non-infected values. A double simultaneous criterion was used to identify differentially expressed genes: i) genes with ratios VHSV-infected/non-infected > 2 and ii) genes which deviated from the null hypothesis using the t-test at p < 0.05. Calculations were made from 4 biological replicates VHSV-infected and non-infected each and by 2 independent researchers using Microsoft Excel, Origin pro SR4, BRB-Array Tools and the TIGR Multiple array viewer program (MeV) (see above) and their results confronted until all discrepancies were solved.

## List of abbreviations

EST: express sequence tag; F-ACTIN: filamentous actin; FFU: focus forming units; *HMGB1*: high mobility group protein 1; HRV: hirame rhabdovirus; IHNV: infectious haematopoietic necrosis virus; KDA: kilo Daltons; MS: mass espectrophotometry; MW: molecular weight; PAGE: polyacrylamide gel electrophoresis; PCR: polymerase chain reaction; PI: isoelectric point; *RPLP0*: ribosomal phosphoprotein p0; RT-Q-PCR: reverse transcriptase quantitative polymerase chain reaction; SDS: sodium dodecyl sulfate; SVCV: spring viremia carp virus; 2D-DIGE: two dimensional differential gel electrophoresis: VHSV: Viral haemorrhagic septicemia virus.

## Authors' contributions

PE and BN performed the RT-Q-PCR and experimental viral infections. MAR and AF carried out the statistical Q-PCR and microarray data analysis. AE and JC design experiments, coordinate the work and drafted-write the manuscript. All authors read and approved the manuscript.
